# Supplementary material for: Terahertz radiation by quantum interference of excitons in a one-dimensional Mott insulator
Source: Nat Commun. 2023 Oct 13;14:6229. doi: 10.1038/s41467-023-41463-8 (PMC10575914; doi:10.1038/s41467-023-41463-8)
Supplement: Supplementary file 1 — Supplementary Information [file 41467_2023_41463_MOESM1_ESM.pdf]

# Supplementary Information:

# Terahertz radiation by quantum interference of excitons in a one-dimensional Mott insulator

Tatsuya Miyamoto[1], Akihiro Kondo[1], Takeshi Inaba[1], Takeshi Morimoto[1], Shijia You[1] and Hiroshi Okamoto[1]

[1]*Department of Advanced Materials Science, University of Tokyo, Chiba 277-8561, Japan*

**Contents:**

**Supplementary Note 1. Details of terahertz radiation experiments**

**Supplementary Note 2. Method for controlling the delay time between the $\omega$-pulse and $2\omega$-pulse**

**Supplementary Note 3. Incident electric-field dependence of terahertz radiation amplitudes**

**Supplementary Note 4. Electric field waveforms of terahertz radiation in non-resonant excitation**

**Supplementary Note 5. Approximation of the third-order nonlinear susceptibility $\chi^{(3)}$**

**Supplementary Note 6. Analyses of $\Delta t$ dependence of the integrated electric field amplitude of terahertz radiation for resonant exciton excitation**

**Supplementary Note 1. Details of terahertz radiation experiments**

Supplementary Figure 1a shows a schematic of the optical system for the terahertz radiation measurements. The output of a Ti: sapphire regenerative amplifier (RA) with a central photon energy of 1.55 eV, a repetition rate of 1 kHz, a pulse duration of 100 fs, and a pulse energy of 2 mJ was divided into two beams using a beam splitter (BS). One of the outputs was used as the input light to an optical parametric amplifier (OPA) to obtain the $\omega$-pulse ($\hbar\omega = 0.64$ eV or 0.585 eV). This pulse was collimated using two parabolic mirrors (PM1 and PM2) and focused onto a second-order nonlinear optical crystal $\beta$-$BaB_2O_4$ ($\beta$-BBO) with a thickness of 1 mm to generate the $2\omega$-pulse ($2\hbar\omega =$ 1.28 eV or 1.17 eV ). The intensity of the $\omega$-pulse was adjusted using a variable neutral density filter (VND). Since most of the intensity of the $\omega$-pulse passed through the $\beta$-BBO crystal, the $\omega$-pulse and $2\omega$-pulse were emitted approximately simultaneously and were parallel and perpendicular to the optical surface plate, respectively. To control the delay time $\Delta t$ of the $\omega$-pulse relative to the $2\omega$-pulse, these two pulses were focused on an $\alpha$-BBO crystal[1]. The details of the method for controlling the delay time $\Delta t$ are reported in Supplementary Note 2. The $b$-axis of a crystal of $[Ni(chxn)_2Br]Br_2$ (chxn= cyclohexanediamine) was tilted by 45° with respect to the two excitation pulses so that both of the pulses have electric-field components along the Ni-Br chain, i.e., the $b$-axis. These polarization configurations are schematically illustrated in Supplementary Fig. 1b. The terahertz waves generated in the reflection configuration were collimated using a parabolic mirror (PM3) and focused onto a 1-mm thick ZnTe crystal using another parabolic mirror (PM4), which is used for electro-optic sampling (EOS)[2]. Two wire-grid polarizers (WG1 and WG2) were inserted between PM3 and PM4 to adjust the polarization direction of the terahertz waves.

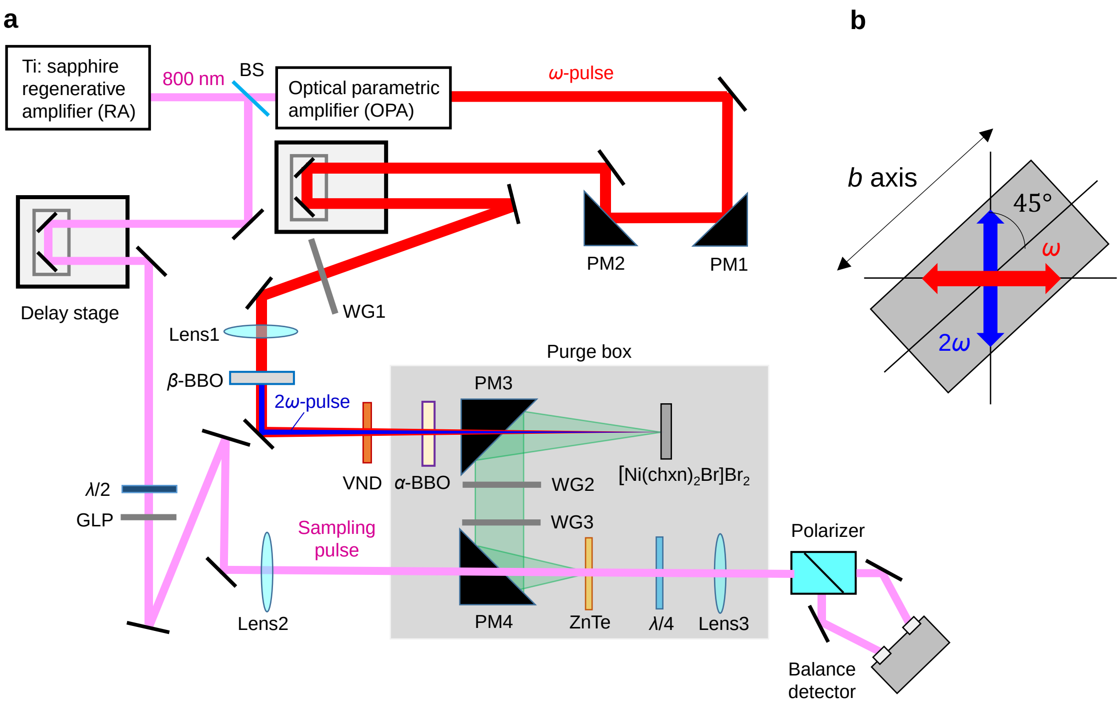


**Supplementary Fig. 1 | Experimental set up of the terahertz radiation measurements.** **a**, The schematic layout of the optical system; WG: wire-grid polarizer, PM: parabolic mirror, VND: variable neutral density filter, $\lambda/2$ ($\lambda/4$): half-wave plate (quarter-wave plate). The optical paths of the $\omega$-pulse and the $2\omega$-pulse are shown by the red and blue lines, respectively. **b**, Polarization directions of the $\omega$-pulse and the $2\omega$-pulse on the measured plane (*bc* plane) of a $[Ni(chxn)_2Br]Br_2$ single crystal.

The other RA output was simultaneously focused through a hole in the parabolic mirror (PM4) onto the ZnTe crystal with the radiated terahertz waves and used as a sampling pulse for the EOS. The polarization direction of WG1 and WG2 was tilted by ±45° and perpendicular to the optical surface plate, respectively, and the sampling pulse was polarized parallel to the optical surface plate. In this configuration, the polarization component of the terahertz waves along the Ni-Br chain (the *b*-axis) can be detected using the EOS. When the polarization direction of WG1 is perpendicular to the Ni-Br chain, terahertz waves were not detected, indicating that the terahertz waves are perfectly

polarized along the Ni-Br chain (the $b$-axis). Electric-field waveforms of the terahertz radiations from ZnTe, Si, and Ge were measured in the same configuration with $\omega = 0.64$ and $2\omega = 1.28$ eV. To suppress the absorption of the terahertz waves due to water vapor in the air, generation and detection were performed in a purge box filled with dry air.

## Supplementary Note 2. Method for controlling the delay time between the $\omega$-pulse and $2\omega$-pulse

In this study, the delay time of the $2\omega$-pulse relative to the $\omega$-pulse, $\Delta t$, was controlled by rotating the $\alpha$-BBO crystal with a thickness $d$ of 490 μm positioned in their optical path as shown in the middle part of Supplementary Fig. 2 (ref. 1). When the angle between the wavenumber vector and the optical axis is $\theta$, the refractive index of an extraordinary ray, $n_e(\theta)$, for a uniaxial crystal such as $\alpha$-BBO and $\beta$-BBO is expressed as follows:

$$n_e(\theta) = \frac{1}{\sqrt{\frac{\cos^2\theta}{n_o^2} + \frac{\sin^2\theta}{n_e^2}}}. \tag{1}$$

where $n_o$ and $n_e$ are the principal refractive indices of the ordinary and extraordinary rays, respectively. The wavelength ($\lambda$) dependence of the refractive index $n(\lambda)$ can be obtained from the Sellmeier equation.

$$n(\lambda) = \sqrt{A + \frac{B}{\lambda^2 - C} - D\lambda^2} \tag{2}$$

The values of $A$, $B$, $C$, and $D$ for $\alpha$-BBO and $\beta$-BBO are shown in Supplementary Table 1. The group refractive index $n_g(\lambda)$ can also be calculated using the relation, $n_g(\lambda) = n(\lambda) - \lambda\frac{dn(\lambda)}{d\lambda}$.

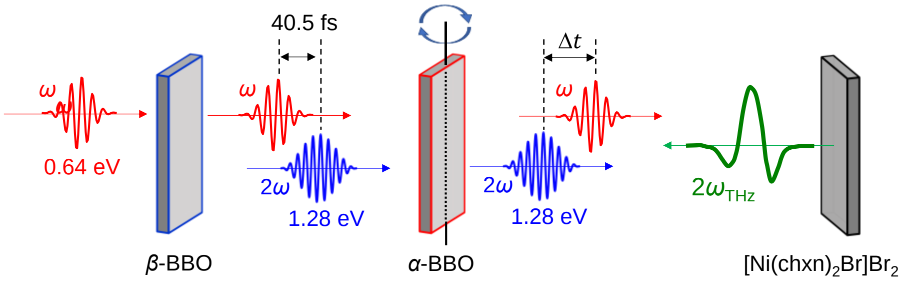


**Supplementary Fig. 2 | The setups of nonlinear optical crystals.** The $\beta$-BBO crystal is used to generate the $2\omega$-pulse from the $\omega$-pulse. The $\alpha$-BBO crystal is used to control the delay time $\Delta t$ of the $\omega$-pulse relative to the $2\omega$-pulse.

**Supplementary Table 1 | Parameters about refractive indices in $\alpha$-BBO and $\beta$-BBO crystals.**

| | | $A$ | $B$ (nm$^2$) | $C$ (nm$^2$) | $D$ (nm$^2$) |
|---|---|---|---|---|---|
| $\alpha$-BBO | $n_o(\lambda)$ | 2.67579 | 0.02099 | 0.00470 | 0.00528 |
| | $n_e(\lambda)$ | 2.31197 | 0.01184 | 0.01607 | 0.00400 |
| $\beta$-BBO | $n_o(\lambda)$ | 2.7359 | 0.01878 | 0.01822 | 0.01354 |
| | $n_e(\lambda)$ | 2.3753 | 0.01224 | 0.01667 | 0.01516 |

As shown in Supplementary Fig. 3, the $\omega$-pulse and $2\omega$-pulse are incident on a $\alpha$-BBO crystal. The incident angle is represented by $\varphi$ and the angle between the normal direction to the crystal's surface and the optical axis of the $\alpha$-BBO crystal is represented by $\alpha$. In the $\alpha$-BBO crystal used in our study, $\alpha = 25°$. In this configuration, the extraordinary ray (the $\omega$-pulse) and the ordinary ray (the $2\omega$-pulse) pass through OA and OB+BC, respectively. Thus, the time delay of the $2\omega$-pulse relative to the $\omega$-pulse, $\delta t$,

occurs in the $\alpha$-BBO crystal, which can be expressed as follows:

$$\delta t = \left(n_{\mathrm{go}}(\lambda_0/2)\frac{\mathrm{OB}}{c} + \frac{\mathrm{BC}}{c}\right) - n_{\mathrm{ge}}(\lambda_0)\frac{\mathrm{OA}}{c}. \tag{3}$$

Here, $n_{\mathrm{go}}$ and $n_{\mathrm{ge}}$ are the group refractive indices of the ordinary and extraordinary rays, respectively. $\lambda_0$ is the wavelength of the $\omega$-pulse, and $c$ is the velocity of light in a vacuum. If the $\varphi$ dependence of $\delta t$ is known, we can control $\delta t$ by rotating the $\alpha$-BBO crystal.

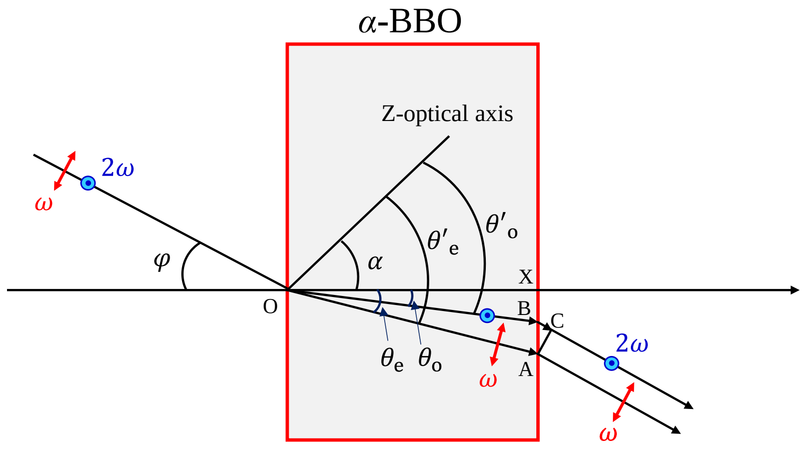


**Supplementary Fig. 3 | Optical paths of the $\omega$- and $2\omega$-pulses in the $\alpha$-BBO crystal.**

The $\varphi$ dependence of $\delta t$ can be deduced in the following way. Here, the refractive angles of the ordinary and extraordinary rays are represented as $\theta_{\mathrm{o}}$ and $\theta_{\mathrm{e}}$ as shown in Supplementary Fig. 3. Using the angles between the optical axis and the optical paths of the ordinary and extraordinary rays, $\theta_{\mathrm{o}}'$ and $\theta_{\mathrm{e}}'$, respectively, $\theta_{\mathrm{o}}$ and $\theta_{\mathrm{e}}$ can be written as $\theta_{\mathrm{o}} = \theta_{\mathrm{o}}' - \alpha$ and $\theta_{\mathrm{e}} = \theta_{\mathrm{e}}' - \alpha$, respectively. From Snell's law, we obtain the following relationships.

$$\sin\varphi = n_{\mathrm{o}}(\lambda_{\mathrm{o}})\sin\theta_{\mathrm{o}} \tag{4}$$

$$\sin\varphi = n_{\mathrm{e}}(\theta_{\mathrm{e}}', \lambda_{\mathrm{e}})\sin(\theta_{\mathrm{e}}' - \alpha) \tag{5}$$

Supplementary Equation (5) can be transformed as follows:

$$\left(\cos^2\alpha - \frac{\sin^2\varphi}{n_{\mathrm{e}}^2(\lambda_{\mathrm{e}})}\right)\tan^2\theta_{\mathrm{e}}' - 2\cos\alpha\sin\alpha\tan\theta_{\mathrm{e}}' + \sin^2\alpha - \frac{\sin^2\varphi}{n_{\mathrm{o}}^2(\lambda_{\mathrm{o}})} = 0. \quad (6)$$

By solving this equation, we obtain $\tan\theta_{\mathrm{e}}'$ as follows:

$$\tan\theta_{\mathrm{e}}' = \frac{\cos\alpha\sin\alpha \pm \frac{\sin\varphi}{n_{\mathrm{e}}(\lambda_{\mathrm{e}})}\sqrt{1 - \frac{\sin^2\varphi}{n_{\mathrm{o}}^2(\lambda_{\mathrm{o}})}}}{\cos^2\alpha - \frac{\sin^2\varphi}{n_e^2}}. \quad (7)$$

Then, $\theta_{\mathrm{e}}$ is described as

$$\theta_{\mathrm{e}} = \arctan\left(\frac{\cos\alpha\sin\alpha \pm \frac{\sin\varphi}{n_{\mathrm{e}}(\lambda_{\mathrm{e}})}\sqrt{1 - \frac{\sin^2\varphi}{n_{\mathrm{o}}^2(\lambda_{\mathrm{o}})}}}{\cos^2\alpha - \frac{\sin^2\varphi}{n_e^2}}\right) - \alpha. \quad (8)$$

The optical path lengths, OA, OB, and BC, can be expressed as follows:

$$\mathrm{OA} = \frac{d}{\cos\theta_{\mathrm{e}}}, \mathrm{OB} = \frac{d}{\cos\theta_{\mathrm{o}}} \text{ and } \mathrm{BC} = d\sin\theta_{\mathrm{e}}\,(\tan\theta_{\mathrm{e}} - \tan\theta_{\mathrm{o}}). \quad (9)$$

Using Supplementary Eqs. (3), (4), (8), and (9), we can calculate the $\varphi$ dependence of $\delta t$, which is plotted in Supplementary Fig. 4 for $\hbar\omega = 0.64$ eV and $\hbar\omega = 0.585$ eV, where $\hbar$ is the reduced Planck constant. When the $\omega$-pulse and $2\omega$-pulse pass through the $\beta$-BBO crystal with $d$ = 1 mm (see the left part in Supplementary Fig. 2), the $2\omega$-pulse precedes the $\omega$-pulse by approximately 40.5 fs for $\hbar\omega = 0.64$ eV (36.3 fs for $\hbar\omega =$ 0.585 eV). Therefore, the actual value of the delay time of the $2\omega$-pulse relative to the $\omega$-pulse, $\Delta t$, is given as $\Delta t = \delta t - 40.5$ fs for $\hbar\omega = 0.64$ eV ($\Delta t = \delta t - 36.3$ fs for $\hbar\omega = 0.585$ eV). Based on these relationships and Supplementary Fig. 4, we can change the actual delay time $\Delta t$ from approximately –12.6 fs to 100.7 fs for $\hbar\omega = 0.64$ eV (–14.2 fs to 98.7 fs for $\hbar\omega = 0.585$ eV) by rotating the angle of the $\alpha$-BBO crystal from

–21.5° to 38.5° using an automated rotation stage.

It should be noted that by rotating the $\alpha$-BBO crystal, not only $\Delta t$, but also the relative time when the generated terahertz wave and the sampling pulse reach the ZnTe crystal used in the EOS are changed. The latter also affects the position of the terahertz waves on the time axis. As such, corrections were implemented when plotting the terahertz electric field waveforms.

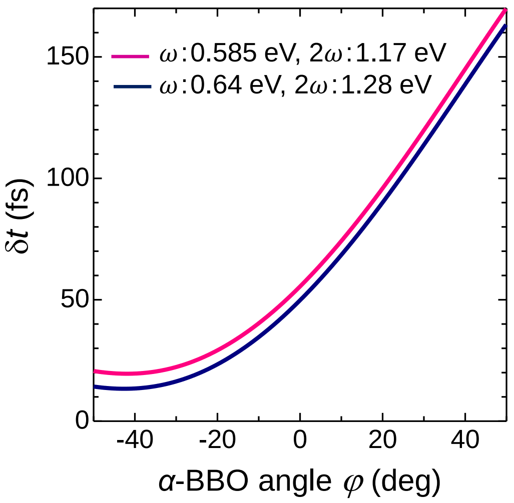


**Supplementary Fig. 4 | The incident angle $\varphi$ dependence of the additional delay time $\delta t$ of the $2\omega$-pulse relative to the $\omega$-pulse in the $\alpha$-BBO crystal.**

## Supplementary Note 3. Incident electric-field dependence of terahertz radiation amplitudes

Supplementary Figure 5 shows the incident-electric-field dependence of the electric-field amplitudes of the terahertz radiation. $E_{\mathrm{THz}}(0)$ is the electric-field amplitude of the terahertz radiation at the time origin measured with $\Delta t = 0.86$ fs. $E_\omega$ and $E_{2\omega}$ are the electric-field amplitudes of the $\omega$-pulse and $2\omega$-pulse, respectively. The data are obtained in the condition of $E_\omega = 8E_{2\omega}$. The broken line shows a relation that $E_{\mathrm{THz}}(0)$ is

proportional to $E_\omega E_\omega E_{2\omega}$. This indicates that the terahertz radiation originates from the third-order nonlinear optical effect.

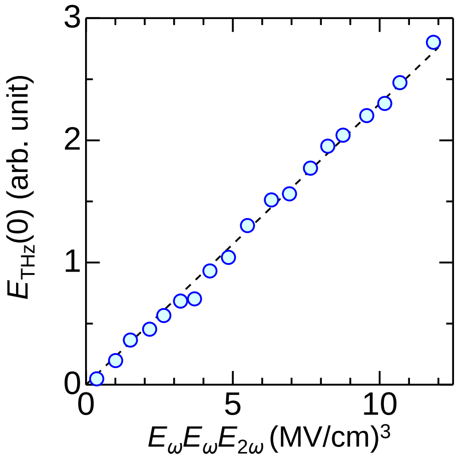


**Supplementary Fig. 5 | Incident-electric-field dependence of the electric-field amplitudes of the terahertz radiation.**

**Supplementary Note 4. Electric field waveforms of terahertz radiation in non-resonant excitation**

In this section, we report on the electric field waveform of the terahertz radiation, $E_{\mathrm{THz}}(t)$. For the non-resonant excitation for the $\omega$-pulse of 0.585 eV and the $2\omega$-pulse of 1.17 eV. Supplementary Figure 6 shows typical results of $E_{\mathrm{THz}}(t)$ at $\Delta t = 0.40$ fs and 78.2 fs in a normalized scale. These waveforms are the same as those at $\Delta t = 0.86$ fs in the resonant excitation with an $\omega$-pulse of 0.64 eV and a $2\omega$-pulse of 1.28 eV, as shown in Fig. 3b in the manuscript. The Fourier amplitude spectra corresponding to these waveforms for resonant and non-resonant excitations are shown in Fig. 5b and 5e, respectively, and are similar to each other as reported in the manuscript.

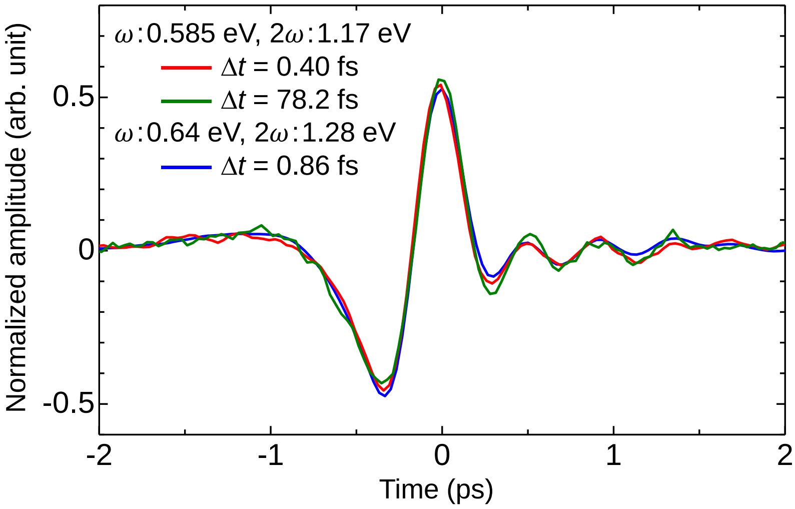


**Supplementary Fig. 6 | Comparison of the electric-field waveforms of the terahertz radiations between resonant and non-resonant excitations.** In the resonant excitation, $\hbar\omega$ = 0.64 eV, $2\hbar\omega$ = 1.28 eV, and $\Delta t$ = 0.86 fs. In the non-resonant excitation, $\hbar\omega$ = 0.585 eV, $2\hbar\omega$ = 1.17 eV, and $\Delta t$ = 0.40 fs or 78.2 fs.

## Supplementary Note 5. Approximation of the third-order nonlinear susceptibility $\chi^{(3)}$

The third-order nonlinear susceptibilities associated with the investigated terahertz radiation are $\chi^{(3)}\left(-\omega_{\mathrm{THz}};\omega,\omega,-(2\omega-\omega_{\mathrm{THz}})\right)$ and $\chi^{(3)}(-\omega_{\mathrm{THz}};-\omega,-\omega,2\omega+\omega_{\mathrm{THz}})$, and their main terms are expressed as follows[3]:

$$\chi^{(3)}_{\mathrm{main}}\left(-\omega_{\mathrm{THz}};\omega,\omega,-(2\omega-\omega_{\mathrm{THz}})\right)$$

$$\approx \frac{Ne^4}{6\varepsilon_0\hbar^3}\frac{\langle 0|x|1\rangle\langle 1|x|2\rangle\langle 2|x|1\rangle\langle 1|x|0\rangle}{(\omega_1-(2\omega-\omega_{\mathrm{THz}})-i\gamma_1)(\omega_2-2\omega-i\gamma_2)(\omega_1-\omega+i\gamma_1)} \tag{10}$$

and

$$\chi^{(3)}_{\mathrm{main}}(-\omega_{\mathrm{THz}};-\omega,-\omega,2\omega+\omega_{\mathrm{THz}})$$

$$\approx \frac{Ne^4}{6\varepsilon_0\hbar^3} \frac{\langle 0|x|1\rangle\langle 1|x|2\rangle\langle 2|x|1\rangle\langle 1|x|0\rangle}{(\omega_1 - (2\omega + \omega_{\mathrm{THz}}) - i\gamma_1)(\omega_2 - 2\omega - i\gamma_2)(\omega_1 - \omega + i\gamma_1)}, \quad (11)$$

where $N$ is the density of the electrons, $\varepsilon_0$ is the permittivity in a vacuum, $e$ is the elementary charge, and $x$ is the displacement of an electron along the 1D axis. In a previous study, the energy of the odd-parity (even-parity) exciton, $\hbar\omega_1$ $(\hbar\omega_2)$, was determined to be 1.27 eV (1.28 eV), and its spectral width, $\hbar\gamma_1$ $(\hbar\gamma_2)$, to be 0.046 eV (0.129 eV) [3]. The transition dipole moments $\langle 0|x|1\rangle$ between the states $|0\rangle$ and $|1\rangle$, and $\langle 1|x|2\rangle$ between the states $|1\rangle$ and $|2\rangle$ were evaluated to be 1.87 Å and 29.9 Å, respectively[4]. In the expressions for $\chi^{(3)}_{\mathrm{main}}$ in Supplementary Eqs. (10) and (11), there is a difference in the denominators, which is $1/(\omega_1 - (2\omega - \omega_{\mathrm{THz}}) - i\gamma_1)$ in Supplementary Eq. (10) and $1/(\omega_1 - (2\omega + \omega_{\mathrm{THz}}) - i\gamma_1)$ in Supplementary Eq. (11). Since $\hbar\gamma_1$ is much larger than $\hbar\omega_{\mathrm{THz}}{\sim}4\,\mathrm{meV}$, we can consider that the following approximation is valid even for resonant excitation with $\hbar\omega = 0.64\,\mathrm{eV}$ and $2\hbar\omega = 1.28\,\mathrm{eV}$.

$$\chi^{(3)}_{\mathrm{main}}(-\omega_{\mathrm{THz}}; \omega, \omega, -(2\omega - \omega_{\mathrm{THz}})) \sim \chi^{(3)}_{\mathrm{main}}(-\omega_{\mathrm{THz}}; -\omega, -\omega, 2\omega + \omega_{\mathrm{THz}}) \quad (12)$$

**Supplementary Note 6. Analyses of $\Delta t$ dependence of the integrated electric field amplitude of terahertz radiation for resonant exciton excitation**

In this section, we report on the analysis of the time characteristic of the integrated electric-field amplitude, $I_{\mathrm{THz}}(\Delta t)$, as shown in Fig. 5c and 5f. As previously indicated, $I_{\mathrm{THz}}(\Delta t)$ includes an oscillatory component owing to a coherent response and a background non-oscillatory component owing to the real exciton response. The envelope of the former oscillatory component is represented by $a(\Delta t)$ and the latter background

component has an envelope given by $b(\Delta t)$. Thus, the envelope of $I_{\mathrm{THz}}(\Delta t)$ is the sum of $a(\Delta t)$ and $b(\Delta t)$.

In the case of non-resonant excitation, $b(\Delta t) = 0$. $a(\Delta t)$ can be expressed using the envelope of the electric field waveform of the $\omega$-pulse, $g_{\omega}(t)$, and that of the $2\omega$-pulse, $g_{2\omega}(t - \Delta t)$ as follows:

$$a(\Delta t) \propto \int_{-\infty}^{\infty} [g_{\omega}(t)]^2 g_{2\omega}(t - \Delta t) dt$$

$$= [g_{\omega}{}^2 \circ g_{2\omega}](\Delta t) \equiv g(\Delta t). \tag{13}$$

The operator $\circ$ represents a cross-correlation function. Assuming that $g_{\omega}(t)$ and $g_{2\omega}(t)$ are Gaussian functions, $g(\Delta t)$ is also a Gaussian function, which is expressed as follows:

$$g(\Delta t) = \frac{1}{\sqrt{\pi}\sigma} \exp\left(-\frac{\Delta t^2}{\sigma^2}\right), \tag{14}$$

where $g(\Delta t)$ corresponds to the instrumental function of the measurement system, and $\sigma$ is a parameter that characterizes the Gaussian function. Its temporal width $t_{\mathrm{w}}$ is $2\sqrt{\ln 2}\sigma$. Using $g(\Delta t)$, the upper envelope of $I_{\mathrm{THz}}(\Delta t)$ in the non-resonant excitation ($2\hbar\omega =$ 1.17 eV) is well reproduced, as shown by the blue line in Fig. 5f. The fitting parameter is $t_{\mathrm{w}}$, which is evaluated to be 120 fs. This is almost equal to the temporal width that is expected from the incident pulses, i.e., approximately 170 fs.

In the case of resonant excitation of excitons ($2\hbar\omega = 1.28$ eV), we should consider the contribution of the real exciton response, $b(\Delta t)$, as well as $a(\Delta t)$. The time characteristics of the phase relaxations of the odd-parity and even-parity excitons dominate $b(\Delta t)$ and are assumed to be expressed by the functions $f_{\mathrm{odd}}(t)$ and $f_{\mathrm{even}}(t)$, respectively:

$$f_{\mathrm{odd}}(t) = \exp\left(-\frac{t}{\tau_{\mathrm{odd}}}\right) u(t) \tag{15a}$$

$$f_{\mathrm{even}}(t) = \exp\left(-\frac{t}{\tau_{\mathrm{even}}}\right)u(t), \tag{15b}$$

where $\tau_{\mathrm{odd}}$ and $\tau_{\mathrm{even}}$ are the time constants of the phase relaxation of the odd-parity and even-parity excitons, respectively, and $u(t)$ is a step function. Using $f_{\mathrm{odd}}(t)$ and $f_{\mathrm{even}}(t)$, $b(\Delta t)$ can be expressed as follows:

$$\begin{aligned} b(\Delta t) &\propto \left[[f_{\mathrm{even}} * g_{\omega}{}^{2}] \circ [f_{\mathrm{odd}} * g_{2\omega}]\right](\Delta t) \\ &= \left[[f_{\mathrm{even}} \circ f_{\mathrm{odd}}] * [g_{\omega}{}^{2} \circ g_{2\omega}]\right](\Delta t) \\ &= \left[[f_{\mathrm{even}} \circ f_{\mathrm{odd}}] * g\right](\Delta t). \end{aligned} \tag{16}$$

The operator $*$ represents the convolution integral. When the excitation of the even-parity excitons by the $\omega$-pulse is preceded by the excitation of the odd-parity excitons by the $2\omega$-pulse ($\Delta t \geq 0$), $[f_1 \circ f_2](\Delta t)$ can be written as follows:

$$\begin{aligned} [f_1 \circ f_2](\Delta t) &= \int_{-\infty}^{\infty} \exp\left[-\left(\frac{1}{\tau_{\mathrm{even}}} + \frac{1}{\tau_{\mathrm{odd}}}\right)t + \frac{\Delta t}{\tau_{\mathrm{odd}}}\right] u(t)u(t-\Delta t)dt \\ &= \left(\frac{\tau_{\mathrm{even}}\tau_{\mathrm{odd}}}{\tau_{\mathrm{even}} + \tau_{\mathrm{odd}}}\right) \exp\left(-\frac{\Delta t}{\tau_{\mathrm{even}}}\right) \end{aligned} \tag{17}$$

Using the expression for $a(\Delta t)$ in Supplementary Eqs. (13) and (14) and that for $b(\Delta t)$ in Supplementary Eqs. (16) and (17), the envelope of $I_{\mathrm{THz}}(\Delta t)$ is also well reproduced by $[a(\Delta t) + b(\Delta t)]$, as shown by the orange line in Fig. 5c. The background component excluding the oscillatory component from $I_{\mathrm{THz}}(\Delta t)$ can be reproduced by $b(\Delta t)$ with $\tau_{\mathrm{even}} = 38$ fs, as shown by the green line in this figure. The time characteristic of $a(\Delta t)$ is expressed with $t_{\mathrm{w}} = 60$ fs, which is shorter compared to the non-resonant excitation condition, $t_w = 120$ fs. This can be explained by the fact that the coherent response is suppressed when the real exciton excitations increase within the temporal width of the laser pulses (see the main text).

In Fig. 5h, we show the evaluated time characteristics of $a(\Delta t)$ for the non-resonant

excitation condition ($2\hbar\omega = 1.17$ eV), and $a(\Delta t)$ and $b(\Delta t)$ for the resonant excitation condition ($2\hbar\omega = 1.28$ eV).

**Supplementary References**


1. Dai, J., Karpowicz, N. & Zhang, X. C. Coherent Polarization Control of Terahertz Waves Generated from Two-Color Laser-Induced Gas Plasma. *Phys. Rev. Lett.* **103**, 023001 (2009).
2. Planken, P. C. M., Nienhuys, H. K., Bakker, H. J. & Wenckebach, T. Measurement and calculation of the orientation dependence of terahertz pulse detection in ZnTe. *J. Opt. Soc. Am. B* **18**, 313–317 (2001).
3. Butcher, P. N. & Cotter, D. *The Elements of Nonlinear Optics* (Cambridge Univ. Press, Cambridge, UK, 1990).
4. Yada, H., Miyamoto, T. & Okamoto, H. Terahertz-field-driven subpicosecond optical switching in a one-dimensional Mott insulators enabled by large third-order optical nonlinearity. *Appl. Phys. Lett.* **102**, 091104 (2013).
